# Estrogenic/Progestin therapy and the development of Vestibular Schwannoma: A systematic review and meta-analysis

**DOI:** 10.1016/j.bjorl.2025.101706

**Published:** 2025-09-25

**Authors:** Maria Vitória Graça Couto de Campos Amaral, Fayez Bahmad Jr

**Affiliations:** Faculdade de Ciências da Saúde da Universidade de Brasília, Brasília, DF, Brazil

**Keywords:** Estrogen, Progestin, Vestibular Schwannoma, Hormone therapy, Meta-analysis

## Abstract

**Objective:**

Compare the risk of developing Vestibular schwannoma in patients prescribed Hormone Therapy (HT).

**Methods:**

We performed a systematic review in accordance with the Preferred Reporting Items for Systematic reviews and Meta-Analysis (PRISMA), the search was conducted in English, and we included all the studies that met the following criteria: (a) Participants who presented with VS; (b) Patients prescribed Hormone Replacement Therapy (HRT); (c) Prospective, retrospective or case-control study.

**Results:**

Of the 146 articles, four met the inclusion criteria and were included in the systematic review and meta-analysis. Two studies documented an increased relative risk of Vestibular schwannoma in patients that had ever used hormone replacement therapy, another study found an incidence rate 2.2 times higher than the expected incidence rate and the other study indicated a 10% increased odds of the Vestibular Schwannoma in patients who have ever used hormone replacement therapy.

**Conclusion:**

The use of hormone replacement therapy was associated with an increased relative risk of Vestibular Schwannoma but also demonstrated a lack of a definitive association.

**Level of evidence:**

Level 1.

## Highlights

• Risk of Vestibular Schwannoma in patients on hormone therapy.
• Hormone therapy linked to higher risk of Vestibular Schwannoma.
• Link between Vestibular Schwannoma and hormone therapy needs more study.

## Introduction

Vestibular Schwannoma (VS), also known as acoustic neuroma, is a benign tumor originating from Schwann cells along the vestibulocochlear nerve, primarily located at the cerebellopontine angle, making up approximately 85% of such cases.^1^ Additionally, it is the most prevalent tumor within the extra-axial posterior fossa compartment among adult patients.^2^

Female sex hormones have been identified as potential influencers of Central Nervous System (CNS) tumor risk, with some reports indicating an elevated risk among users of menopausal Hormone Therapy (HT), although the evidence remains limited.^3^ Notably, the use of specific HT for menopause has shown varying effects on the risk of different cancer types, including increased risks associated with breast, ovarian, and endometrial cancers, and decreased risks linked to gastrointestinal tract cancers. However, the association between HT use and the risk of specific CNS tumors is an ongoing area of investigation.^4^

In the realm of CNS tumors, which are relatively rare, only a limited number of prospective studies have explored the relationship between Hormone Replacement Therapy (HRT) and tumor risk. These studies have generated mixed results, with one reporting a significant increase in meningioma risk among current HRT users, while another found no significant association concerning the risk of glioma.^5^^,^^6^

Our systematic review has the primary objective to compare the risk of developing Vestibular schwannoma in women prescribed Hormone Therapy (HT).

## Methods

### Search strategy

The search strategy was conducted in English and article selection was performed by searching PubMed, MedLine, and Livivo. There was no restriction on the language of publication and no restriction on the period of publication. The following terms were used to identify original articles and reviews: (vestibular schwannoma) AND (“estrogen replacement” or “oestrogen replacement” or “estrogen” or “estrogen therapy” or “oestrogen” or “oestrogen therapy” or “estrogen replacement therapy” or “oestrogen replacement therapy” or “hormone” or “hormone replacement therapy” or “hormone therapy” or “hormonal therapy” or “hormonal replacement therapy”). This same search strategy was used in all databases. The initial query identified 146 articles that were subsequently screened by a thorough review of the article titles, abstracts, and manuscripts, as necessary. A manual search of references of retrieved articles was performed to identify additional relevant publications.

The protocol was registered at the international prospective register of systematic reviews (PROSPERO) under registration number CRD42024519734.

### Eligibility criteria

Studies were included if they were investigating the occurrence of Vestibular Schwannoma (VS) in patients prescribed Hormone Replacement Therapy (HRT). We included all the studies that met the following criteria: (a) Participants who presented with VS; (b) Patients prescribed Hormone Replacement Therapy (HRT); (c) Prospective, retrospective or case-control study. Additionally, the following exclusion criteria were applied: (1) Studies involving animals; (2) Single-blind, or non-controlled trials; (3) Case reports; (4) Insufficient data on previous/current hormonal replacement therapy. Two reviewers conducted all phases of the study selection. During the first phase, the reviewers assessed the titles and abstracts of the studies to determine their suitability.

### Data extraction

For the meta-analysis, the data collection process was performed independently by the same 2 reviewers. Any discordances would be solved through discussion. The extracted data from each study included: author, year of the study, study design, number of participants, HT usage, risk estimates and corresponding 95% Confidence Intervals (95% CIs). The data collection was performed independently by the same 2 reviewers.

Effect sizes from individual studies were synthesized to calculate pooled estimates of Relative Risk (RR), Standardized Incidence Ratio (SIR) and Odds Ratio (OR), along with their corresponding 95% CIs. Forest plots were generated to visually represent effect sizes and confidence intervals from individual studies, along with overall pooled estimates. These graphs also highlighted the statistical significance of each study's findings, with p-values noted next to the effect sizes.

All statistical analyzes were performed using Python software version 3.12 (2023). Disparities were resolved by discussion.

## Results

### Study selection

**Fig. 1:**
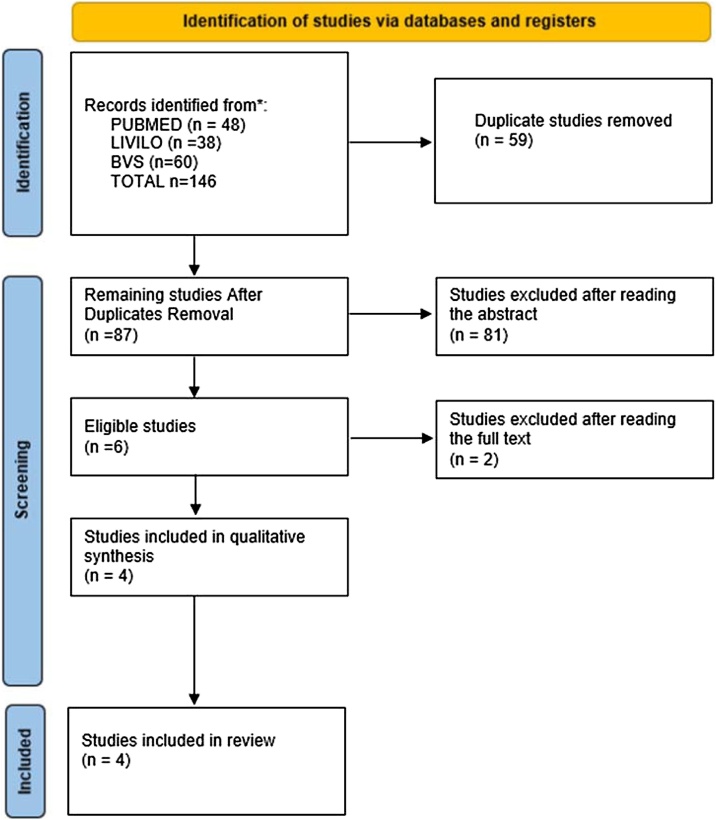
Flow diagram of study selection modified from PRISMA.

This systematic review was performed according to the Preferred Reporting Items for Systematic reviews and Meta-Analysis (PRISMA) criteria. The initial database searches identified 146 articles in three databases. After duplicates removal 87 articles were remaining. Of the 87 remaining articles, 81 were excluded by the established exclusion criteria. The most common reasons for exclusion were histopathological studies (n = 29), case reports (n = 14). After this, there were six articles remaining for the second phase, which consisted of full text analysis. Two articles were excluded in this stage because the information on the use of hormonal drugs was not included. After the exclusion, there are 4 studies remaining included in this review. Of the 4 studies, three contain information related to the occurrence of VS in patients prescribed Hormone Replacement Therapy (HRT) and one compares the incidence of VS in transgender individuals receiving cross-sex hormone treatment (Fig. 1).

### Study characteristics

Schoemaker et al. is a comprehensive population-based case-control study across Nordic countries and the United Kingdom, that investigated the etiology of acoustic neuroma, specifically exploring its correlation with various medical factors. Analyzing 291 cases of vestibular schwannoma and 1449 controls, the study searched into elements such as medical history, cigarette smoking, allergy diagnosis, antiallergenic medication use, epilepsy history, neoplasm diagnosis, head injury, birth characteristics, and female sex hormone-related factors. Notably, the research revealed no significant association between acoustic neuroma risk and the use of oral contraceptives (OR = 0.9, 95% CI 0.7–1.3), hormone replacement therapy (OR = 1.1, 95% CI 0.8–1.6), or long-acting hormonal contraceptives. However, it's crucial to highlight that the study did not provide specific details regarding the number of cases and controls associated with hormonal replacement therapy, warranting further investigation into this aspect.

Benson et al. is a prospective and meta-analysis study published in 2015 in which collected information on HT prescriptions in women aged 50–79 years with CNS tumors diagnosed in 1987–2011. The number of CNS tumors was 3.500 of which 439 are VS. This study found a significantly increased risk of developing acoustic neuroma in wome prescribed HT (RR = 1.37 [95% CI 1.06–1.75]). The design and the characteristics of the study population are published by Million Women Study Collaborative Group.

Benson et al. is another prospective study published in 2010 with 1,147,894 postmenopausal women, exploring the relationship between Hormone Replacement Therapy (HRT) and the occurrence of Central Nervous System (CNS) tumors. Among the participants, there were 117 cases of acoustic neuromas. Compared to women who had never used HRT, the Relative Risks (RRs) for all incident CNS tumors in those currently using HRT were 1.58 (95% CI 1.02–2.45). For women who were current users of oestrogen-only HRT, there were 22 cases, and the relative risk was 1.94 (1.15–3.29). On the other hand, for those using oestrogen-progestagen HRT, there were 16 cases, and the relative risk was 1.13 (0.63–2.05). This study found an increased risk of developing acoustic neuroma in woman prescribed HT (RR = 1.64 [95% CI 1.11–2.41]). This finding also suggests that the present use of oestrogen-only HRT might elevate the likelihood of acoustic neuroma.

Nota et al. is a study that explored the potential influence of hormonal agents on benign brain tumors, this study investigated the risk in transgender individuals undergoing cross-sex hormone treatment, focusing on a cohort of 2555 transwomen and 1373 transmen. Out of 20 identified benign brain tumor cases, the breakdown included two vestibular schwannomas. The study encompassed various hormone regimens, for transwoman it included oestradiol implants, oral oestradiol valerate, oestradiol patches, and oestradiol gel. Notably, the analysis revealed no significant difference in the occurrence of vestibular schwannomas (n = 2; SIR = 2.2, 95% CI 0.4–7.3, where SIR stands for standardized incidence ratio) compared to the expected numbers in the general female and male populations. Notably, both cases of vestibular schwannomas diagnosed under cross-sex hormone treatment involved Tibolon 2.5 mg or CPA 20 mg/week and oestradiol patch 100 ug, with Gonadectomy (GDX). However, it's crucial to acknowledge that the data regarding trans men were not included in this systematic review, warranting that they received testosterone-based treatment.

### Characteristics of the included studies

Table 1.

**Table 1:** Characteristics of the included studies.

Study	Author. Year	Study design	Sample VS	Population	No HT prescription	Current/Previous HT prescription	Risk Estimates	95% CI
Medical history, cigarette smoking and risk of acoustic neuroma: An international case-control study	Schoemaker et al. 2007^8^	Case-Control	291	Cisgender Woman	ND	ND	OR = 1.1	0.8–1.6
Menopausal hormone therapy and central nervous system tumor risk: Large UK prospective study and meta-analysis.	Benson et al. 2015 ^3^	Prospective	439	Cisgender Woman	286	153	RR = 1.37	1.06–1.75
Hormone replacement therapy and incidence of central nervous system tumours in the Million Women Study.	Benson et al. 2010^7^	Prospective	117	Cisgender Woman	40	77	RR = 1.64	1.11–2.41
The occurrence of benign brain tumours in transgender individuals during cross-sex hormone treatment	Nota et al. 2018^9^	Retrospective	2	Transgender Woman	DNA	2	SIR = 2.2	0.4–7.3

HT, Hormonal Therapy; ND, Not Described; DNA, Does Not Apply; CI, Confidence Interval; RR, Relative Risk; OR, Odds Ratio; SIR, Standardized Incidence Ratio.

#### The risk of developing acoustic neuromas in women prescribed hormone therapy

**Fig. 2:**
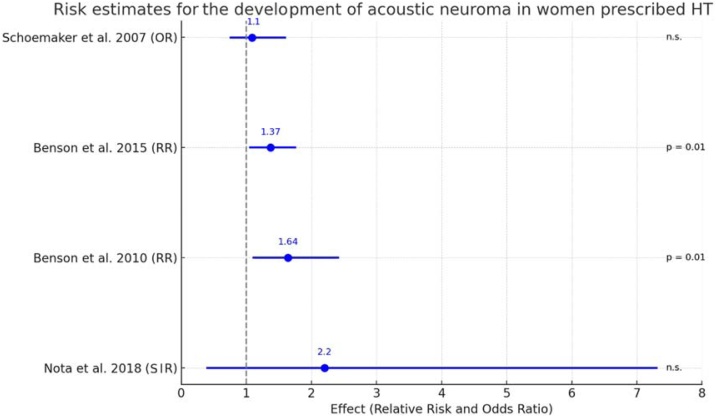
Forest graph showing for four studies the values of association strength by Relative Risk (RR), Odds Ratio (OR) and Standardized Incidence Ratio (SIR) with their respective confidence intervals and statistical significance. The vertical dashed line shows the point of nullity of association. n.s, not significant.

The forest plot illustrates the comparative effect sizes and statistical significance of four studies within a meta-analysis, focusing on Relative Risk (RR), Odds Ratio (OR) and Standardized Incidence Ratio (SIR) as measures of association. Benson et al., 2014 and Benson et al., 2010 report Relative Risk with estimates of 1.37 (95% CI 1.06–1.75) and 1.64 (95% CI 1.11–2.41), respectively, both reaching statistical significance with p-values of 0.01. The confidence intervals of these two studies do not exceed the null value (RR = 1), reinforcing the strength of these associations (Fig. 2).

Schoemaker et al., 2006, reporting an Odds Ratio of 1.1 (95% CI 0.8–1.6), does not reach statistical significance, indicated by the term “n.s.” (not significant). The confidence interval for this study includes the null value (OR = 1), indicating uncertainty about the direction and magnitude of the effect (Fig. 2).

Nota et al., 2018 presents an SIR result of 2.2, although not significant, with a large confidence interval that goes from 0.4 to 7.3, which demonstrates great variability in the data obtained by the author and the possibility of nullity risk between cases and controls due to the scope of the value 1.0.

The visual representation through colored points and lines (blue for RR and green for OR and SIR) together with the placement of significance values increase the clarity of the results, allowing an understanding of effect sizes and their statistical relevance. The alignment of studies from Benson et al. on the side of increased risk (greater than 1) with significant p-values highlights a potential association between exposure and outcome. In contrast, Schoemaker et al. and Nota et al. positions and its wide-ranging confidence interval crossing the null illustrate the lack of a definitive association (Fig. 2).

### Risk of bias

Each included study was analyzed according to the Joanna Briggs Institute risk of bias assessment using the JBI “Critical Appraisal Checklist” for Case Control and Cohort studies. In all 4 articles, after judgment, two studies showed a low risk of bias^3^^,^^7^ and two articles showed a moderate risk of bias.^8^^,^^9^

### Quality assessment

The studies that assessed occurrence of VS in patients prescribed Hormone Replacement Therapy (HRT) were evaluated by the NEWCASTLE ‒ OTTAWA Quality Assessment Scale for Cohort and Case Control Studies (NOS). The NOS comprises eight criteria assessing patient selection, study comparability and outcome/exposure of interest. Ratings are assigned by awarding points for high-quality elements, and the total points are used for quantitative comparison of study quality. The maximum score is 9 points, reflecting the highest methodological quality. We adopted the Newcastle-Ottawa Scale to evaluate research quality and defined them as high, middle, and low quality by score 7–9, 4–6, 1–3, respectively. Any discrepancies were resolved through collaborative reevaluation of the original article by discussion (Table 2).

**Table 2:** Newcastle-Ottawa scale scores of the included studies.

	Selection (4 stars)				Comparability (2 stars)	Exposure (3 stars)			Total score
Case Control Studies	Case Definition	Representativeness of cases	Selection of Controls	Definition of Controls		Assessment of exposure	Same method of ascertainment for cases and controls	Non-Response Rate	
Schoemaker^8^	★	★	★	★	‒	★	★	★	7

Cohort Studies	Representativeness of the exposed cohort	Selection of the non-exposed cohort	Ascertainment of exposure	Demonstration that outcome of interest was not present at start of study		Assessment of outcome	Was follow up long enough for outcomes to occur?	Adequacy of follow up of cohort	
Benson^3^	★	★	★	★	★	★	★	★	8
Benson^7^	★	★	★	★	★	★	★	★	8
Nota^10^	★	★	★	★		★	★	★	7

‒, The data was not available.

## Discussion

In this study, we used data from four studies comprising 849 patients to conduct a systematic review and meta-analysis and examine the association between HRT use and the risk VS, after combining the results from two prospective studies, one case control study and one retrospective study. Our analysis shows that half of all studies found a significantly elevated risk of VS in association with HRT.^3^, ^4^, ^5^, ^6^, ^7^ We found that the average relative risk was 1.505 (95% CI), this risk should be interpreted with caution considering that it only takes into account two out of the four including studies.

The increase in VS risk among users of oestrogen-only HRT was documented by Benson et al. 2010 with an RR of 1.94 (1.15–3.29).

The remaining two studies are not able to be taken into account for relative risk due to the heterogeneity of risk estimates used by the authors. In contrast, one study indicated a 10% increased odds of acoustic neuroma in ever having used hormone replacement therapy but it is not statistically significant.^8^ The other observed an incidence rate of 2.2 times higher than the expected incidence rate,^9^ although not significant, due to uncertainty in the estimate and the possibility of nullity of risk between cases and controls.

The potential limitations of our study should be considered when interpreting the results. However, based on the broad spectrum of studies identified, we believe that this review is a comprehensive and representative assessment of the current state of knowledge regarding this topic. In the future, the association between VS and HRT use should be tested in further studies.

## Conclusion

In conclusion, this meta-analysis showed that the use of HRT was associated with an increased relative risk of VS.

## ORCID ID

Maria Vitória Graça Couto de Campos Amaral: 0009-0002-8755-2934

## Funding

The author(s) received no financial support for the research, authorship, and/or publication of this article.

## Declaration of competing interest

The author(s) declared no potential conflicts of interest with respect to the research, authorship, and/or publication of this article.
